# Clinical risk factors for preeclampsia in twin pregnancies

**DOI:** 10.1371/journal.pone.0249555

**Published:** 2021-04-15

**Authors:** Nipp Chantanahom, Vorapong Phupong

**Affiliations:** Department of Obstetrics and Gynecology, Faculty of Medicine, Chulalongkorn University, Bangkok, Thailand; University of Insubria, ITALY

## Abstract

**Background:**

Preeclampsia is a common obstetric complication. The rate of preeclampsia is increased in twin pregnancies. The aim of this study was to assess the clinical risk factors for developing preeclampsia in twin pregnancies.

**Methods:**

A case-control study was carried out among women with twin pregnancies who delivered at gestational age more than 23 weeks at King Chulalongkorn Memorial Hospital, Department of Obstetrics and Gynecology, Faculty of Medicine, Chulalongkorn University, Bangkok, Thailand, from 2003 to 2019. The data were retrieved from electronic medical records. Multivariate logistic regression analysis was used to find the risk factors.

**Results:**

A total of 1,568 twin pregnancies were delivered during the study period and 182 cases (11.6%) developed preeclampsia. 172 cases with preeclampsia and 516 controls were selected for analysis. After certain variables were adjusted in the multivariate logistic regression analysis, the clinical factors associated with preeclampsia in twin pregnancies were nulliparity (adjusted odds ratio (OR) 1.57, 95% confidence interval (CI) 1.02–2.41) and chronic hypertension (adjusted OR 6.22, 95%CI 1.98–19.57). Low gestational weight gain was a significant protective factor against the development of preeclampsia (adjusted OR 0.50; 95%CI 0.32–0.77).

**Conclusion:**

The clinical risk factors for developing preeclampsia in twin pregnancies were nulliparity and chronic hypertension. These risk factors are of value to identify twin pregnant women at risk for preeclampsia and in implementing primary prevention.

## Introduction

Hypertensive disorders in pregnancy constitute one of the leading causes of maternal and perinatal mortality worldwide. Estimated global prevalence is around 2–8% of pregnancies. Currently, there are various risk factors that increase the probability of preeclampsia and a multifetal gestation is one of the risk factors [1].

In twin pregnancies, the rate of preeclampsia is higher than singleton and overall rate is around 9.5%, about two- to three-fold increased risk compared to singleton [2]. Furthermore, preeclampsia in twins has been reported to occur at earlier gestational age and has more severe form [3]. The reasons why twins developed preeclampsia more than singleton are still inconclusive. However, it has been proposed that the pathogenesis of preeclampsia among twin pregnancies may be due to a higher immunologic response and placental mass [4].

There are few studies that assessed the risk factors for preeclampsia in twins and have conflicting results [5–8]. Furthermore, some clinical risk factors were not included in their research. One previous study conducted in Asians did not find any associated risk factors for developing preeclampsia in twins [7]. Although various retrospective studies found that nulliparity, black race, and in-vitro fertilization (IVF) might increase the risks, however, in one study, the data was retrieved from birth certificates whereas in another study, the old diagnostic criteria of preeclampsia was used [9, 10]. One previous study found risk factors for hypertensive disorders including gestational hypertension and preeclampsia [8]. Therefore, the aim of this study was to assess the clinical risk factors for preeclampsia in twin pregnancies.

## Materials and methods

This was a case-control study carried out at the Department of Obstetrics and Gynecology, King Chulalongkorn Memorial Hospital, Faculty of Medicine, Chulalongkorn University, Bangkok, Thailand. The research protocol was approved by the Institutional Review Board of the Faculty of Medicine, Chulalongkorn University. The ethics committee waived the need for informed consent to have data from the medical records used in this study.

All obstetrics and neonatal records of twin deliveries at > 23 weeks gestations and resulted in two live births from January 2003 to December 2019 were reviewed. Exclusion criteria were gestational hypertension, pre-existing renal disease, and hydrops fetalis in at least one fetus. Demographic data, clinical characteristics, and delivery outcomes were extracted for analysis.

Preeclampsia was defined as having a blood pressure of ≥ 140/90 mmHg or more on two occasions at least 4 hours apart accompanied by proteinuria (≥ 300 mg/dl of protein in a 24-hour urine collection, or a urine protein-to-creatinine ratio ≥ 0.3, or ≥ 1+ on urine dipstick) after 20 weeks of gestation [11]. Severe preeclampsia was defined as having a blood pressure of ≥ 160/100 mmHg at least 4 hours apart when the patient is on bed rest accompanied by thrombocytopenia, impaired liver function, severe persistent right upper quadrant or epigastric pain, progressive renal insufficiency, pulmonary edema, or new-onset cerebral or visual disturbances [11].

Clinical risk factors that were evaluated in this study were nulliparity, elderly gravidarum, late antenatal care, IVF, egg donation, obesity, gestational weight gain, previous preeclampsia, aspirin use during antenatal care, chronic hypertension, pre-gestational diabetes, and gestational diabetes.

Gestational diabetes was diagnosed according to Carpenter and Coustan or National Diabetes Data Group (NDDG) criteria [12], and pre-gestational diabetes was diagnosed using the standard diagnostic criteria of hemoglobin A_1C_ (HbA_1C_) ≥ 6.5%, a fasting plasma glucose of ≥ 126 mg/dl, or a 2-hour glucose of ≥ 200 mg/dl or greater on a 75-g oral glucose tolerance test [13].

Pre-pregnancy body mass index (BMI) (kg/m^2^) was calculated and organized into 4 categories using the Asia-Pacific criteria [14]: underweight (BMI < 18.5 kg/m^2^), normal weight (BMI 18.5–22.9 kg/m^2^), overweight (BMI 23–24.9 kg/m^2^), and obese (BMI ≥ 25kg/m^2^). Gestational weight gain during pregnancy was stratified according to the Institute of Medicine 2009 recommendations [15]. The gestational weight gain was correlated to Asia-Pacific BMI criteria. The gestational weight gain corrected for the gestational age at delivery.

Chorionicity was determined by standard first or second trimester ultrasounds based on the number of gestational sacs, yolk sacs, amniotic sacs, placental locations, membranes’ thickness, λ-sign, and T-sign. Twins of different sexes were considered to be dichorion. The chorionicity was confirmed with operative findings and available pathological reports.

The primary objective was to identify the risks for developing preeclampsia in twins. The secondary objective was to compare the neonatal outcomes between preeclampsia and the control groups.

The sample size was calculated based on the risk factors from a previous study [6]. Pre-pregnancy obesity was the risk factor that gave the largest sample size. Thus, the sample size of 172 twin cases with preeclampsia and 516 controls were needed to detect a statistical difference (α = 0.05, β = 0.1, and case -to-control ratio 1: 3). Cases of twins with preeclampsia were randomly selected from the hospital database. Controls were every 3 cases of twins without preeclampsia who admitted consecutively after preeclamptic women.

Statistical analysis was carried out by SPSS software version 22.0. Categorical variables were presented as frequency and percentages, whereas continuous variables were presented as mean and standard deviation. Chi-square test and Fisher-exact test were used to compare categorical variables and student’s t-test was used for continuous variables as appropriate. Variables with a p value < 0.05 in the univariate analysis were entered into the multivariate logistic regression model. The variables: nulliparity, chronic hypertension, gestational weight gain, and body mass index were added in multivariate logistic regression. Odds ratio (OR) and 95% confidence interval (CI) were calculated. P value < 0.05 was considered statistically significant.

## Results

During the study period, a total of 1,568 twin deliveries met the inclusion criteria of which 182 twins (11.6%) developed preeclampsia. 146 cases (80.2%) had late-onset preeclampsia and 36 cases (19.8%) had early-onset preeclampsia. 97 cases (53.3%) had severe feature and 85 cases (46.7%) had non-severe feature.

The demographic data of the study population are presented in Table 1. Of these 172 preeclamptic women, 91 cases had severe feature and 81 cases had non-severe feature. There was significantly more proportion of women with chronic hypertension in the preeclampsia group. Pre-pregnancy bodyweight, body mass index (BMI), gestational weight gain, systolic blood pressure, and diastolic blood pressure during antenatal care were also significantly greater in twins with preeclampsia than without preeclampsia. Cesarean section rate was higher and hospital stays were longer in the preeclampsia group. There was no case of pre-gestational diabetes in both groups.

**Table 1:** Demographic characteristics of the study population.

Characteristics	No pre-eclampsia (*n* = 516)	Pre-eclampsia (*n* = 172)	P value
Age (years)	30.9 ± 5.7	31.1 ± 6.2	0.822
Gravida			0.403
• Primigravida	245 (47.5%)	88 (51.2%)	
• Multigravida	271 (52.5%)	84 (48.8%)	
Parity			0.041
• Nulliparity	309 (59.9%)	118 (68.6%)	
• Multiparity	207 (40.1%)	54 (31.4%)	
Previous abortion	112 (21.7%)	45 (26.2%)	0.26
Pregestational diabetes	0	0	NA
Chronic hypertension	5 (1.0%)	12 (7.0%)	< 0.001
Smoking	2 (0.4%)	1 (0.6%)	1.000
IVF	84 (16.3%)	38 (22.1%)	0.083
Gestational age at first ANC (weeks)	13.3 ± 6.1	12.6 ± 4.9	0.171
Late ANC	211 (40.9%)	64 (37.2%)	0.393
Number of ANC	8.1 ± 2.8	7.8 ± 2.6	0.153
Pre-pregnancy bodyweight (kg)	54.5 ± 10.3	57.9 ± 12.5	0.001
BMI (kg/m^2^)	21.7 ± 3.8	22.7 ± 4.5	0.01
< 18.5 kg/m^2^	88 (17%)	26 (15.1%)	0.005
18.5–22.9 kg/m^2^	282 (54.7%)	72 (41.9%)	
23–24.9 kg/m^2^	64 (12.4%)	37 (21.5%)	
≥ 25 kg/m^2^	82 (15.9%)	37 (21.5%)	
Total weight gain (kg)	16.3 ± 5.9	19.0 ± 8.7	< 0.001
SBP during ANC (mmHg)	111.9 ± 9.9	119.9 ± 11.9	< 0.001
DBP during ANC (mmHg)	69.8 ± 8.4	73.7 ± 10.9	< 0.001
Aspirin use	16 (3.1%)	4 (2.3%)	0.601
Chorionicity			0.111
Dichorion	291 (56.4%)	85 (49.4%)	
Monochorion	225 (43.6%)	87 (50.6%)	
Route of delivery			
Cesarean section	439 (85.1%)	157 (91.3%)	0.038
Other routes	77 (14.9%)	15 (8.7%)	
Length of hospital stays (days)	6.9 ± 8.1	8.2 ± 5.8	0.022

Data are shown as mean ± SD, or as n (%).

OR: odds ratio, CI: confidence interval, IVF: in-vitro fertilization, BMI: body mass index, ANC: antenatal care, SBP: systolic blood pressure, DBP: diastolic blood pressure.

In the univariate analysis (Table 2), the significant risk factors for preeclampsia were nulliparity, chronic hypertension, and excessive gestational weight gain, while low gestational weight gain was a significant protective factor for preeclampsia.

**Table 2:** Risk factors for preeclampsia in twin pregnancies from univariate analysis.

Risk Factor	No preeclampsia (n = 516)	Preeclampsia (n = 172)	OR (95% CI)
Nulliparity	309 (59.6%)	118 (68.6%)	1.46 (1.01–2.11)
Elderly gravidarum	151 (29.2%)	53 (30.8%)	1.08 (0.73–1.59)
Previous preeclampsia	1 (0.2%)	2 (1.2%)	6.06 (0.55–67.24)
Aspirin use during ANC	16 (3.1%)	4 (2.3%)	0.75 (0.25–2.27)
IVF	84 (16.3%)	38 (22.2%)	1.47 (0.95–2.25)
Egg donation	1 (0.2%)	2 (1.2%)	6.06 (0.55–67.24)
Chronic hypertension	5 (1.0%)	12 (7.0%)	7.66 (2.66–22.09)
Gestational diabetes	31 (6.0%)	10 (5.8%)	0.97 (0.43–2.11)
Late ANC	211 (40.9%)	64 (37.2%)	0.86 (0.59–1.24)
Obesity	82 (15.9%)	37 (21.5%)	1.45 (0.92–2.29)
Excessive gestational weight gain	33 (6.4%)	21 (12.2%)	2.04 (1.10–3.75)
Low gestational weight gain	206 (39.9%)	46 (26.7%)	0.55 (0.37–0.82)
Monochorionicity	225 (43.6%)	87 (50.6%)	1.32 (0.92–1.90)

OR: odds ratio, CI: confidence interval, ANC: antenatal care, IVF: in-vitro fertilization

The results from the multivariate logistic regression analysis are shown in Table 3. Independent risk factors significantly associated with preeclampsia in the twins were nulliparity (adjusted OR 1.57; 95%CI 1.02–2.41), and chronic hypertension (adjusted OR 6.22; 95%CI 1.98–19.57). On the contrary, low gestational weight gain was a significant protective factor for preeclampsia (adjusted OR 0.50; 95%CI 0.32–0.77).

**Table 3:** Results from the multivariate logistic regression analysis.

Risk Factor	Adjusted OR^a^ (95% CI)
Nulliparity	1.57 (1.02–2.41)
Chronic hypertension	6.22 (1.98–19.57)
Low gestational weight gain	0.50 (0.32–0.77)

OR: odds ratio, CI: confidence interval

^a^Adjusted for nulliparity, chronic hypertension, gestational weight gain, and body mass index

Neonatal outcomes are shown in Table 4. Aside from having a significant higher rate of preterm delivery, transient tachypnea of newborn and lower mean birth weights in the preeclampsia group, there were no other significant differences in neonatal outcomes between the two groups.

**Table 4:** Comparison of the neonatal outcomes.

Neonatal Outcome	No preeclampsia (newborn = 1032)	Preeclampsia (newborn = 344)	P value
Gestational age at delivery (weeks)	35.3 ± 2.5	35.5 ± 5.4	0.461
Preterm	614 (59.5%)	240 (69.8%)	< 0.001
Term	418 (40.5%)	104 (30.2%)	
Birth weight (grams)	2221.2 ± 490.0	2140.0 ± 484.2	0.008
< 1500 grams	91 (8.8%)	38 (11%)	
1500–2500 grams	647 (62.7%)	227 (66%)	0.068
> 2500 grams	294 (28.5%)	79 (23%)	
Indication for cesarean section			
Fetal distress/ Non-reassuring fetal status	50 (4.8%)	16 (4.7%)	0.884
Apgar < 7 at 1 minute	94 (3.3%)	32 (9.3%)	0.914
Apgar < 7 at 5 minutes	19 (1.8%)	4 (1.2%)	0.543
Neonatal complications			
RDS	102 (9.9%)	26 (7.6%)	0.198
TTNB	66 (6.4%)	9 (2.6%)	0.011
IVH	14 (1.4%)	4 (1.2%)	1.000
NEC	10 (1.0%)	6 (1.7%)	0.383
Sepsis	69 (6.7%)	14 (4.1%)	0.077
NICU admission	132 (12.8%)	41 (11.9%)	0.672

Data are shown as mean ± SD, or as n (%).

RDS: respiratory distress syndrome, TTNB: transient tachypnea of newborn, IVH: intraventricular hemorrhage, NEC: necrotizing enterocolitis, NICU: neonatal intensive care unit

## Discussion

This study demonstrated that nulliparity and chronic hypertension were significantly associated with increased risk for developing preeclampsia in twins, while low gestational weight gain was a significant protective factor against preeclampsia.

The risk factors for developing preeclampsia in twins in this study were different from previous studies [5–7]. This may be due to the differences in the ethnicity of the study population studied and clinical risk factors that were studied. Fox et al found egg donation and prepregnancy obesity were independent risk factors for preeclampsia in twin pregnancies [6]. Their study was conducted in Caucasians and excluded chronic hypertension and monochorionic monoamniotic twins. Lučovnik et al’s study was conducted in Slovenia and found high pre-pregnancy BMI and gestational diabetes were significantly associated with preeclampsia in twin gestation [5]. But, Suzuki and Igarashi did not find any significant risk factors for developing preeclampsia in Japanese twin pregnancies [7].

For this study, nulliparity was one of the significant risk factors for preeclampsia in twin pregnancies. This was similar to a previous population-based study in twins [9]. This could be explained by the immunologic response of the mother’s tolerance towards the paternal derived fetal and the placental antigen was lower in nulliparous women [16]. This risk factor was also a significant risk for preeclampsia in a study that included singleton and multifetal pregnancies [17].

In addition, for this study, chronic hypertension was a significant risk factor for preeclampsia in twin pregnancies. Likewise, previous studies that included singleton and multifetal pregnancies also reported that chronic hypertension was a risk factor for preeclampsia [18, 19]. The reason for this is that chronic hypertension can cause end organs damage and vascular complications.

Chorionicity was not found to be associated with increased risk for developing preeclampsia in twin pregnancies in this study. This finding is in line with the results previously reported [20, 21]. They found similar rate of preeclampsia in dichorionic and monochorionic twins [20, 21]. This finding was inconsistent with previous studies by Campbell et al [22] and Spark et al [23]. Campbell et al found a higher rate of preeclampsia in monochorionic twins [22]. There is still no pathophysiologic explanation behind this association. It was postulated that this may be associated with altered birth weight, placental size, or the relationship between them [22]. Spark et al demonstrated that dichorionic twins had higher odds of developing preeclampsia without severe features [23].

Regarding gestational weight gain, for this study, excessive gestational weight gain was not a risk factor for preeclampsia in twin pregnancies after adjusting for confounding variables. This was inconsistent with previous studies that found excessive gestational weight gain contributed to higher rates of preeclampsia in twin pregnancies [24, 25]. This difference may be due to the ethnicity. Low gestational weight gain was a protective factor against the development of preeclampsia in this study. This was similar to a previous study that low gestational weight gain decreased the risk of preeclampsia with an OR of 0.48 (0.24–0.97) [25].

The strengths in our study were its large sample size and all relevant clinical risk factors were collected for analyses. This study was limited by its retrospective nature. However, a case-control design was appropriate to find the risk factors in this study. The diagnostic criteria for severe preeclampsia here differs slightly from the diagnostic criteria by ACOG. This difference was the limitation of the study. Other limitations were that we did not include twin with single fetal demise and there were few patients with egg donation and history of previous preeclampsia. Moreover, there was no pre-gestational diabetes in both groups of participants, thus the effect of this factor on preeclampsia in twins could not be determined.

## Conclusions

This study demonstrated that significant risk factors for preeclampsia in twins were nulliparity and chronic hypertension, while low gestational weight gain was a significant protective factor for preeclampsia in twin pregnancies. These risk factors are of value to identify twin pregnant women at risk for preeclampsia and in implementing primary prevention.
